# The language of all medical publications and spine publications from 1950 to 2020^☆^

**DOI:** 10.1016/j.xnsj.2022.100118

**Published:** 2022-04-21

**Authors:** Nicolas Pascual-Leone, Jennifer W. Liu, Alexander Beschloss, Srish S. Chenna, Comron Saifi

**Affiliations:** a Perelman School of Medicine at the University of Pennsylvania, United States; b Houston Methodist Hospital, Department of Orthopaedic Surgery, United States; c University of Pennsylvania, School of Engineering and Applied Science, United States

## Abstract

**Background:**

Excellent research in all fields, including spine surgery, exists in many different regions and languages. This study seeks to determine the relative number of spine related peer-reviewed publications throughout the world based on language.

**Methods:**

Peer-reviewed publications from the eleven most prolific languages in regard to both the number of peer-reviewed spine publications indexed in PubMed and total peer-reviewed publications from 1950-2020 were identified in PubMed.

**Results:**

29,711,547 peer-reviewed publications were analyzed for the languages of interest with 870,404 (3.0%) of those being spine related peer-reviewed publications. Between 1988 and 2019, non-English language peer-reviewed publications decreased annually for both all peer-reviewed publications and spine related peer-reviewed publications by 44% and 36%, respectively. All medical and spine specific peer reviewed publications in English compared to non-English publications have increased by 7.22 and 6.35 times since 1988, respectively. While the ratio of non-English to English spine related publications decreased in all eleven countries, the percentage of the number of spine specific publications written in Chinese (462%), Portuguese (378%), and Spanish (88%) have increased by the listed percentages.

**Conclusion:**

While the proportion of peer-reviewed publications in the field of spine surgery written in English have increased over the past several decades, there are many non-English language peer-reviewed publications each year, particularly in Chinese. Although the rapid increase in the proportion of English spine related publications is beneficial to English speaking physicians and researchers, further research is necessary to understand the impact on non-English speaking physicians and researchers.

## Introduction

The number of non-English journals and their impact is declining within many scientific fields [1]. Currently, most of the influential medical journals worldwide are written in the English language [2], [3], [4]. From the 1880s to the early 2000s, medical journals written in English in Index Medicus/Medline rose from 35% to 89% while other languages saw a significant decrease, for example, German went from 25% to 1.9% [3].

When scientific breakthroughs are not published in the English language, newfound knowledge remains inaccessible to English-only speaking scientists. This impasse leads to ‘lost’ science which may cause delays in potential advances in a field. By elucidating and developing a strong understanding of the knowledge present within the realm of ‘lost’ science, specifically in the field of spine research, there exists the potential to improve patient-outcomes worldwide.

## Materials and methods

An in-depth literature review was conducted using PubMed to identify all peer-reviewed publications based on the language they were written in. The search results were filtered to isolate articles from 1950-2020. All data analysis using Microsoft Excel Version 16.16.18 for macOS.

The languages in this study were selected based on the total number of peer-reviewed publications in PubMed. The top 20 non-English languages publishing in PubMed were found using the search algorithm: (“INSERT LANGUAGE HERE”[Language]). The top 20 were further analyzed by the number of peer-reviewed publications related to the spine using the following PubMed search algorithm: (“INSERT LANGUAGE HERE”[Language]) AND ((spine) OR (disc herniation) OR (radiculopathy) OR (spondylolisthesis) OR (spinal) OR (scoliosis) OR (myelopathy) OR (kyphosis) OR (lordosis) OR ('pelvic incidence') OR (Odontoid) OR ('degenerative disc disease') OR (lamina) OR (vertebral) OR (vertebrae) OR (vertebra) OR (pedicle) OR ('spinous process') OR ('transverse process')). Peer-reviewed publications yielded from this search will be referred to as “spine publications.” Ultimately, the top 10 non-English languages with peer-reviewed publications in spine were selected: Chinese, Czech, French, German, Italian, Japanese, Polish, Portuguese, Russian, and Spanish. (“INSERT LANGUAGE HERE”[Language]) was used to query for all peer-reviewed publications in a specific language.

Peer-reviewed publications were separated by year of publication between 1950-2020. Individual languages were analyzed based on total number of publications, number of spine publications, and percentage of publications. Further analysis of spine data included calculations of percent spine publications relative to all peer-reviewed publications for a given year. Ultimately, the publication data for all non-English languages were combined and examined relative to the publication data for the English language.

## Results

Between 1950 and 2020, a total of 29,711,547 peer-reviewed publications were analyzed for the languages of interest (Chinese, Czech, English, French, German, Italian, Japanese, Polish, Portuguese, Russian, and Spanish), with 870,404 (3.0%) of those being spine publications. As shown in Table 1, 87% of spine publications were published in English, while 12.9% were published in non-English languages. The next 10 most common languages of spine publications in order were German, French, Japanese, Russian, Chinese, Spanish, Italian, Polish, Czech, and Portuguese. Spine publications relative to all publications in a given language was found to be around 2-3% in all languages analyzed, with Japanese and Chinese having the highest (3.7%) percent of spine publications relative to all publications. Since 1988, there has been a 306% increase in spine publications written in English while there has been a 36% decrease in the spine publication written in non-English languages. There has been a decrease in non-English language spine publications in all languages except for Chinese, Spanish, and Portuguese. All medical publications have also seen a similar trend with increased rates of publication in English, Chinese, Portuguese, and Spanish, and decreased rates of publication in all other languages analyzed.

**Table 1:** Summary of spine publications and all medical publications broken down by language from 1950-2020. The “Non-English” language row includes aggregate data from all 10 Non-English languages studied.

Language	Spine Publications 1950-2019	All Publications 1950-2019	Spine Publications Relative to All Publications	Percent Change in Spine Publications Since 1988	Percent Change in All Publications Since 1988
English	758,532 (87.2%)	25,685,456 (86.5%)	3.0%	306%	303%
Non-English:	111,872 (12.9%)	4,026,091 (13.6%)	2.8%	-36%	-44%
German	23,097 (2.7%)	856,791 (2.9%)	2.7%	-63%	-48%
French	21,514 (2.5%)	717,725 (2.4%)	3.0%	-77%	-59%
Japanese	15,594 (1.8%)	426,769 (1.4%)	3.7%	-59%	-77%
Russian	14,736 (1.7%)	693,636 (2.3%)	2.1%	-75%	-84%
Chinese	11,809 (1.4%)	322,106 (1.1%)	3.7%	462%	228%
Spanish	8,883 (1.0%)	345,902 (1.2%)	2.6%	88%	48%
Italian	7,724 (0.9%)	297,932 (1.0%)	2.6%	-96%	-88%
Polish	4,545 (0.5%)	172,824 (0.6%)	2.6%	-85%	-90%
Czech	2,113 (0.2%)	88,893 (0.3%)	2.4%	-69%	-86%
Portuguese	1,857 (0.2%)	103,513 (0.4%)	1.8%	378%	209%

**Fig. 1:**
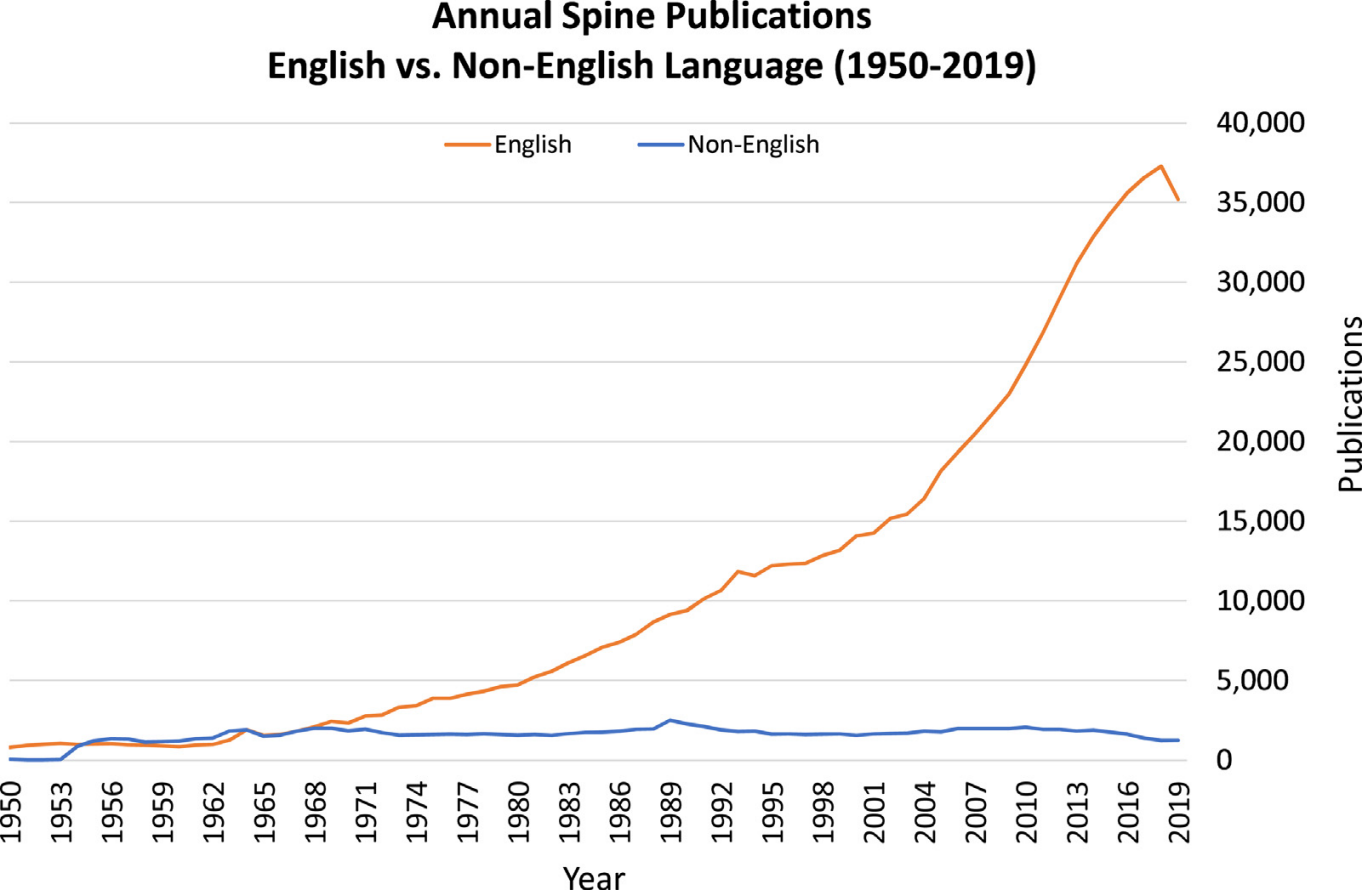
Annual number of spine publications from 1950-2019 in English vs. Non-English languages.

The trends for annual number of English and non-English spine publications from 1950 to 2019 are shown in Fig. 1. From 1950 to 1964 the number of spine publications written in English and non-English languages were comparable. However, since 1964 there has been a massive increase in the annual number of spine publications written in English, with a 1792% increase in English spine publications from 1964 to 2019. During this same time period the annual number of non-English spine publications has roughly stayed the same, with a 34% decrease from 1964 to 2019.

**Fig. 2:**
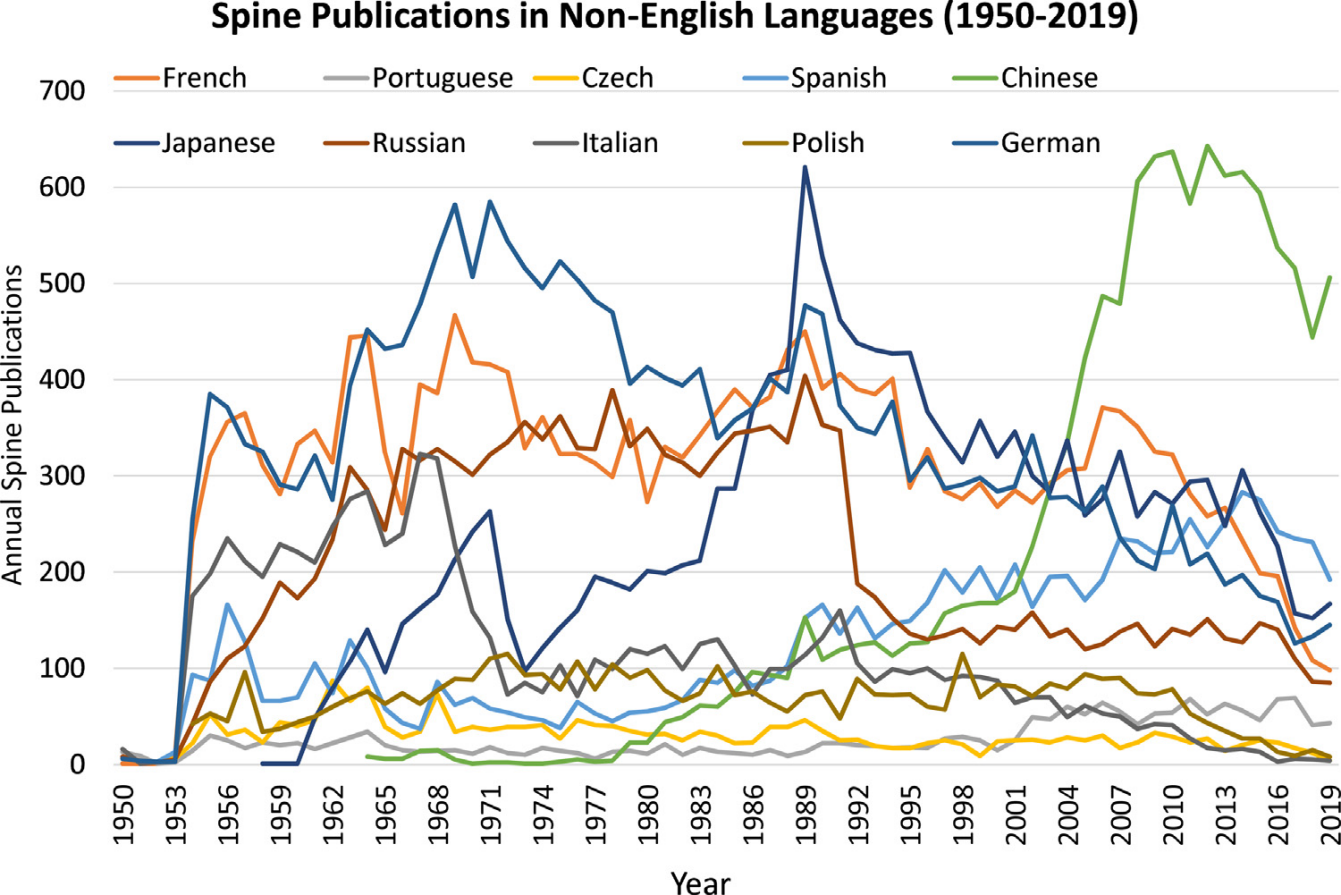
Spine publications broken down by individual non-English language from 1950-2019.

When looking at spine publications in non-English languages, from the years 1950-2000, German, French, Russian, and Japanese were generally the most common non-English language of publication. However, around the early 2000s, Chinese jumped to become the most common non-English language for spine publications. (Fig. 2).

## Discussion

**Fig. 3:**
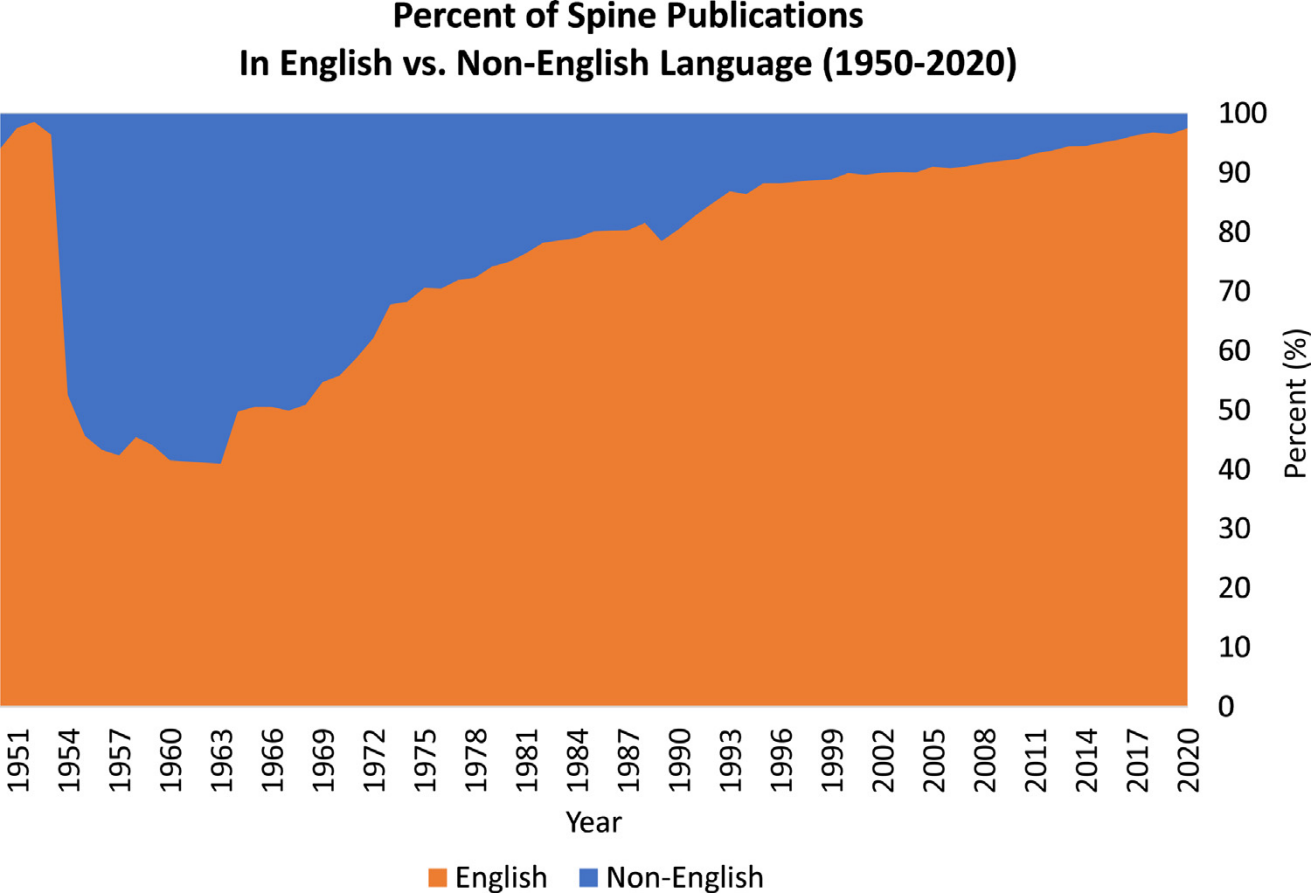
Percent of spine publications in English vs. Non-English languages between the years of 1950-2020.

This study investigates the scale of ‘lost’ science that exists across countries and language in the field of spine surgery. Given the ease of access to information in the modern age, research accessibility depends more on the language of publication than on the country of origin. Between 1950 and 1953, 96% of peer-reviewed publications were in English. Soon after, peer-reviewed publications began to appear in non-English languages. In the early 1960s, the majority of peer-reviewed publications related to the spine were in non-English languages. However, over the next three decades, peer-reviewed publications on spine were increasingly published in English (Fig. 3). English peer-reviewed spine publications make up 87% of spine related peer reviewed peer-reviewed publications between 1950 and 2020. Since 1988, however, peer-reviewed spine publications written in Chinese have increased by 462% - a rate 1.51 times greater than that seen in English peer-reviewed spine publications (306%) during this interval. Portuguese and Spanish, with increases by 378% and 88%, respectively, join Chinese and English as the only languages analyzed which had an increasing number of peer-reviewed spine publications since 1988 (Table 1). The overall 36% decrease in non-English peer-reviewed spine publications, and a 306% increase in English peer-reviewed spine publications suggest countries may be publishing in English in efforts to champion English as a universal language of scientific research.

With 111,872 peer-reviewed spine publications since 1950 having been written in a non-English language, there is a huge volume of peer-reviewed spine publications that are otherwise inaccessible to English-only speaking scientists and surgeons; yet, based on the rate of increasing number of peer-reviewed spine publications in English, it seems that there has been an international effort to make English the new universal language. However, with data showing that some languages like Chinese, Portuguese, and Spanish are continuing to increase their spine publications, may suggest that international spine research might be slower to adopt English as a universal language than the rest of the scientific community. This is merely a suggestion and requires further investigation to determine the cause of this discrepancy. These studies may seek to focus on the allocation of funds towards various scientific fields in these countries specifically. Furthermore, the observed trends in both all and spine-focused peer-reviewed publications stratified by language do indeed require further investigation to determine their causes.

Clearly, this investigation has shown that there is a massive number of peer-reviewed spine publications that are inaccessible to English-only speaking spine scientists and surgeons. It also reveals that there are 758,532 peer-reviewed spine publications written in English that are not accessible to non-English speaking spine surgeons. If addressed, this could help disseminate peer-reviewed research both in the United States and across the globe which may increase the body of knowledge available to clinicians and possibly translate to improved patient outcomes.

Compared to English (3.0%), non-English languages (2.8%) published a lower percentage of their total peer-reviewed publications on the spine since 1950. Individually however, the percent of total peer-reviewed publications that were published on spine in French (3.0%), Chinese (3.7%) and Japanese (3.7%) was greater than that of English. These languages were also found to contribute relatively more to spine than to all fields (Table 1). While the languages of English and Chinese continue to focus on peer-reviewed spine publications by increasing the number of peer-reviewed spine publications, Portuguese, and Spanish were the only other languages found to be increasing peer-reviewed spine publications since 1988. Further investigation as to the cause of these trends is needed to truly understand why these languages are preferentially publishing in spine research while many others are not. Focus on funding resources and grant acceptances for spine research may help elucidate the trends of relative research in various fields. Nonetheless, despite the increase in English peer-reviewed publications, with four different languages increasing the number of peer-reviewed spine publications, ‘lost’ peer-reviewed spine publications will continue to exist and efforts to gain access to all peer-reviewed spine publications are needed.

In 2008, the majority of scientific peer-reviewed publications coming out of Brazil were still in Portuguese. However, they were mostly covering topics of peripheral interests and had low impact factors and limited peer-review [5]. Thus, the Scientific Electronic Library Online (SciELO) was created to publish in an open-access model some of the best Brazilian journals. This gained traction throughout Latin America quickly and currently contains over 1200 journals. Resources like these have made great strides to establish high quality research in non-English speaking countries and provide an excellent resource for many physicians, but it does not provide a solution to bridging this gap [6].

The aforementioned low representation of neurosurgical research in Latin America represents an example of ‘lost’ science, however, language barriers are bi-directional. While the ‘lost’ science in this case usually refers to the spine research that English-only speaking surgeons do not have access to, many non-English speaking spine surgeons may not have access to the vast volume of English-written spine research. This gap has much potential to represent the true ‘lost’ science in the world of spine surgery.

Spinal pathology is a global issue and therefore bridging scientific knowledge across countries and languages is crucial. Low back pain is the leading cause of disability worldwide [7], [8], [9], [10]. In addition, Pellise et al. found that the global burden of adult spinal deformity was not only massive, but had lower quality of life scores than diabetes, chronic lung disease, congestive heart failure and arthritis [11]. Furthermore, considering that spinal fusion and laminectomy with excision of disc, when combined, were the 3^rd^ most common inpatient surgical procedure in 2014, one can begin to understand the scale of impact that spine-related pathology has on patients both in the United States (US) and globally [12]. International spine research may yield innovation to benefit spine surgeons and their patients. For example, a recent study from Lin et al. from the Department of Orthopedic Surgery, China Medical University Hospital, suggests a new strategy for treating infectious spondylodiscitis through minimally invasive endoscopic techniques [13]. There are also many discoveries in the field of spine surgery that are written in English, that would conversely be inaccessible to non-English speaking surgeons. Limiting these knowledge gaps across countries and languages could serve to help physicians and patients alike.

57% of peer-reviewed publications on the spine are from non-US countries and 13% of peer-reviewed publications on the spine have been published in non-English languages. The patients of spine surgeons are from diverse backgrounds and cultures. Recent work done by Jiang et al. and Hines et al. focused on quality improvement in the field of spinal surgery [14, 15]. Jiang et al. suggest that reporting of outcomes has remained inconsistent within the field, while Hines et al. note that the use of registries will help deliver patient centered, efficacious care [14, 15]. Reporting outcomes across all languages to such registries will create massive databases that physicians will then be able to access and further evaluate how certain treatments will affect their patients. The reason that improving the accessibility of this research is because the relative underreporting of outcomes due to physical or language barriers will increase ‘lost’ data and thus result in suboptimal care [14].

We believe significant efforts should be made to disseminate the ‘lost’ science. Current efforts are being made by some journals, like the Journal of International Medical Research, to provide a translation service for select languages. While these efforts are appreciated, greater efforts need to be made for all languages. Increasing the number of languages covered by these translation services would be an example of addressing this problem. Unfortunately, in an ever growing and maturing field, medical terminology can be difficult to translate. Efforts to create specific translational services for medical literature could be the key to producing reliable translations.

This present study has limitations. For example, our method of retrieving peer-reviewed publications does not account for every published article. PubMed provides an excellent database of many of the top journals. Most of the ‘lost’ science is not appearing in these top journals and therefore, if anything, use of PubMed increases the likelihood of obtaining peer-reviewed publications from the United States and in English, while leaving those peer-reviewed publications from lesser-known journals and thus underestimates the degree of contributions from other countries and languages.

## Conclusion

Our data suggests that while English continues to contribute the most to the field of spinal surgery, the historic volume of peer-reviewed publications in other languages and different countries is massive and less accessible to American Spine Surgeons. Furthermore, the vast volume of English peer-reviewed spine publications are inaccessible to non-English speaking spine researchers and surgeons. Since 1988, there have been powerful surges in the number of written peer-reviewed publications on spine in the languages of English and Chinese and decreases in nearly all other investigated languages. This suggests that the languages of English and Chinese may one day be the most prevalent, creating a knowledge barrier between those who speak only one, or neither of those languages. As the field of spine research continues to expand globally, limiting the extent of these bi-directional ‘lost’ findings will be key in providing optimal care to our patients. Addressing this discrepancy will be vital to ensure that both National and International spine-related care improves such that patients across the world benefit from relevant research, no matter the language of origin.

## Declarations of Competing Interests

One or more authors declare potential competing financial interests or personal relationships as specified on required ICMJE-NASSJ Disclosure Forms.
